# Public Perspectives of Oral and Maxillofacial Injuries Related to Domestic Abuse Experiences and Help‐Seeking Barriers: Web Scraping of Reddit Posts

**DOI:** 10.1111/edt.70011

**Published:** 2025-08-13

**Authors:** Corinne Berger, Ana Beatriz Cantao, Noemie N'Diaye, Gustavo Teixeira Bittencourt de Oliveira, Liran Levin

**Affiliations:** ^1^ Department of Surgery, College of Medicine University of Saskatchewan Saskatoon Saskatchewan Canada; ^2^ College of Dentistry University of Saskatchewan Saskatoon Saskatchewan Canada; ^3^ College of Pharmacy and Nutrition University of Saskatchewan Saskatoon Saskatchewan Canada; ^4^ University Center of Para Institute of Computer Engineering Belem Para Brazil

**Keywords:** bruise, head, inter‐personal violence, neck, tooth injury

## Abstract

**Background:**

Domestic abuse (DA) frequently results in injuries to the head, neck, and orofacial regions. Despite the visibility of these injuries, many survivors do not access formal medical or dental care because of fear, stigma, or systemic barriers. Reddit, an anonymous online platform, offers a unique opportunity to examine unfiltered victims/survivors' narratives shared in public forums. The aim of this study was to explore how DA, particularly its physical, psychological, and social impacts, was represented, perceived, and discussed on Reddit. Special attention was given to posts describing injuries to the head, neck, and orofacial region, to understand how victims/survivors narrated their experiences, sought support, and navigated disclosure in anonymous digital spaces.

**Methods:**

This study employed web scraping to analyze Reddit posts from four domestic abuse‐related subreddits (r/AbuseInterrupted, r/DomesticAbuse, r/DomesticViolence, and r/domesticviolence) using Python's Reddit API Wrapper (PRAW). Posts were filtered using anatomical keywords relevant to dental and maxillofacial trauma. After cleaning and manual review, first‐person accounts referencing injuries to the head, neck, or orofacial area underwent qualitative thematic analysis and quantitative content analysis.

**Results:**

A total of 588 Reddit posts related to DA were initially collected. Of the 588 posts, 153 (26.0%) met the inclusion criteria and were retained for analysis. Analysis of the 153 posts meeting the inclusion criteria revealed the most affected regions in DA victims, with frequent descriptions of physical abuse including slapping, grabbing, strangulation, and blunt‐force trauma. Thematic analysis identified four central themes: (1) visible injuries, (2) barriers to accessing medical and dental care, (3) psychological and emotional consequences of abuse, and (4) inconsistent responses from healthcare and legal systems.

**Conclusions:**

Oral and Maxillofacial injuries may serve as critical red flags of domestic abuse. Even when visible, they are often overlooked by healthcare providers. The findings of this study underscore the need for trauma‐informed training among dental professionals and support the integration of domestic abuse screening protocols into routine oral health care. Additionally, the ethical use of web scraping presents a valuable tool for public health research by amplifying survivor voices and helping to identify intervention gaps that may be missed in clinical or institutional data.

## 1 Introduction

Domestic abuse (DA) encompasses intimate partner violence (IPV), family abuse, and other forms of coercive control and is a pervasive global public health issue [1, 2]. It affects individuals across all demographic categories, with higher prevalence among women, children, elders, and LGBTQI+ communities, and manifests in various forms, such as physical, emotional, sexual, and economic abuse [1, 2, 3]. Although DA can leave deep psychological marks, its physical manifestations are often visible, particularly through injuries localized to the head, neck, and orofacial region [4, 5, 6].

Physical harm in DA incidents often affects the dental and maxillofacial regions, making these areas especially vulnerable to trauma. Injuries in these regions commonly include bruises, facial fractures, jaw dislocations, broken or avulsed teeth, lip lacerations, and eye trauma, forms of violence that not only cause acute pain and visible disfigurement but also leave lasting psychological consequences [7, 8, 9]. These physical manifestations of abuse are often associated with significant psychosocial consequences, such as shame, embarrassment, and social withdrawal, that may persist long after the injuries have healed [10, 11, 12]. Studies indicate that a substantial proportion of physical abuse involves trauma to the head, face, and orofacial region [4]. The relationship between maxillofacial injuries and different forms of interpersonal violence, including child maltreatment, youth violence, intimate partner violence, and elder abuse, is well established in the literature, drawing attention to the importance of dental professionals in the early recognition and appropriate response to such cases [5, 8, 13].

However, despite the high visibility and diagnostic value of these injuries, many survivors do not access formal healthcare services. Fear of retaliation, dependency on the abuser, lack of trust in authorities, and concerns about child custody or legal consequences often deter victims from seeking help [14, 15, 16]. Compared to other forms of interpersonal violence, DA may pose a high risk, particularly when survivors attempt to seek help or disclose abuse, as abusers may escalate control in response to perceived threats to their dominance [17]. This dynamic is particularly acute in marginalized populations, such as racialized individuals, transgender people, women, and older adults [15, 18], who face layered barriers including discrimination, inadequate institutional support, and social invisibility. As a result, formal healthcare systems may miss critical opportunities for intervention, especially in cases where physical injuries might otherwise serve as clear clinical indicators of abuse.

Reddit is a popular web‐based social media platform that allows users with registered accounts to submit, share, and engage in discussions across a wide range of topics. All content must be posted within specific subreddits—user‐curated and moderated communities organized around particular themes, interests, or identities. The structured and thematic nature of these subreddits has attracted considerable interest from researchers, as they offer a valuable environment for in‐depth exploration of focused subjects. Reddit is particularly relevant for studies involving sensitive issues, such as DA, because it includes subreddits explicitly dedicated to survivor support, personal disclosure, and community dialogue. These spaces offer public access to organically generated narratives without the ethical complications of infiltrating private or closed online groups, where consent and privacy concerns may limit research access [19, 20].

In this context, digital platforms like Reddit have emerged as alternative disclosure, coping, and community‐building spaces. Reddit hosts a variety of support‐oriented forums (subreddits), many of which allow anonymous users to share long‐form narratives. These digital spaces provide a low‐barrier environment where victims/survivors can speak freely without fear of surveillance, judgment, or forced disclosure. Prior studies have shown that Reddit can be a valuable resource for identifying health‐related patterns and exploring stigmatized topics that are often hidden in formal data systems [20, 21, 22].

Importantly, discussions on Reddit may contain detailed descriptions of injury types and contexts of abuse, offering a unique opportunity to understand how victims/survivors narrate, contextualize, and seek validation or support for the abuse and physical and psychological traumas. These discussions may also reveal how different communities, such as women, children, elders, and LGBTQI+ individuals, experience, interpret, and articulate violence and get help differently in online spaces [19, 23].

The aim of this study was to explore how DA, particularly its physical, psychological, and social impacts, was represented, perceived, and discussed on Reddit. Special attention was given to posts describing injuries to the head, neck, and orofacial region, to understand how victims/survivors narrated their experiences, sought support, and navigated disclosure in anonymous digital spaces. The study looked into understanding how victims/survivors expressed their experiences of violence, the barriers they encountered in accessing formal support systems, and the challenges they faced when seeking support.

## 2 Methods

This study analyzed publicly accessible Reddit posts to explore references to dental and maxillofacial injuries within the context of DA. The study was exempt from review and approval by the Research Ethics Board (REB; E‐Bio‐060), as the study involved analysis of publicly available anonymized online content. No direct interaction with Reddit users occurred, and all data were de‐identified to maintain confidentiality.

Reddit was selected as the data source because of its structure of topic‐specific communities, or “subreddits,” where users often share detailed narratives about personal experiences. The research process began with the identification of suitable online communities where individuals discuss experiences with DA: r/AbuseInterrupted, r/DomesticAbuse, r/DomesticViolence, and r/domesticviolence. Only publicly accessible posts were considered; no private or restricted forums were accessed. Data were collected from the first available Reddit post in these communities up to January 2025.

To collect the data, Python's Reddit API Wrapper (PRAW) was used to programmatically access Reddit's public API. Posts were scraped from each of the four subreddits using keyword filtering. The predefined keywords targeted specific anatomical references relevant to the study's focus: *head, neck, face, nose, mouth, lips, forehead, teeth, and tooth*. Posts containing any of these terms were extracted along with metadata such as the post title, full‐text content, timestamp, and number of comments.

Following data collection, the initial dataset underwent preprocessing to ensure accuracy and relevance. This included the removal of duplicate entries, the exclusion of non‐English posts, and filtering out spam or bot‐generated content. The cleaned data were exported into a structured Excel file to support manual review and content analysis.

Three independent reviewers screened each post for relevance on the basis of the following inclusion criteria: (1) The post must describe physical injury to one or more of the specified anatomical areas: *jaw, face, eye, ears, nose, tongue, teeth, mouth, lips, throat, neck, forehead, hair, head*. (2) The experience must be described from the perspective of the original poster (first‐person account).

Exclusion criteria: (1) absence of reference to physical injury; (2) injuries limited to unrelated body regions; or (3) descriptions of others' experiences without self‐reference. Discrepancies among reviewers were resolved through group discussions until a consensus was reached, ensuring the accuracy and consistency of the final dataset.

A content analysis was conducted to systematically examine the anatomical focus of the physical acts described in the dataset. Mentions of body parts were identified, coded, and counted. Singular and plural forms (e.g., eye/eyes and tooth/teeth) were consolidated under a single category for consistency. Synonymous or anatomically related terms (e.g., lips and mouth) were coded separately to maintain anatomical specificity.

The dataset was further analyzed for the presence of physical aggression, which appeared in various morphological forms, such as base (e.g., *slap*), past tense (e.g., *slapped*), and present participle (e.g., *slapping*). Common verbs included grab, slap, strangle, shove, smack, pull, choke, and bash. Some verbs were grouped by meaning (e.g., strangle/choke) for analysis. These action words vary in grammatical form but also reflect differing degrees of force and intent, from less severe acts like push or shove to more severe ones like slam, bash, or strangle.

To analyze thematic patterns within the narratives, a qualitative thematic analysis was conducted on the final set of included posts. The research team employed an inductive approach to coding, allowing themes to emerge from the data without a predetermined framework. Each post was read in full and coded manually by the researchers, who then collaboratively grouped the codes into broader themes through discussion and iterative refinement.

## 3 Results

A total of 588 Reddit posts related to DA were initially collected. Of the 588 posts, 153 (26.0%) met the inclusion criteria and were retained for analysis. Posts were excluded (*n* = 427; 72.6%) if they lacked relevant anatomical references or were not written in the first person. Although some posts contained figurative expressions involving anatomical terms (e.g., “It's a real kick in the teeth”), these were not counted as a separate category, as they did not meet the inclusion criteria requiring explicit reference to physical injury. Retained posts were long and descriptive, with a mean character length of 263 compared to 211 for excluded posts. Upvotes varied widely (Mean = 27.6, SD = 34.5), with no significant difference in upvote distribution between included and excluded posts.

**FIGURE 1:**
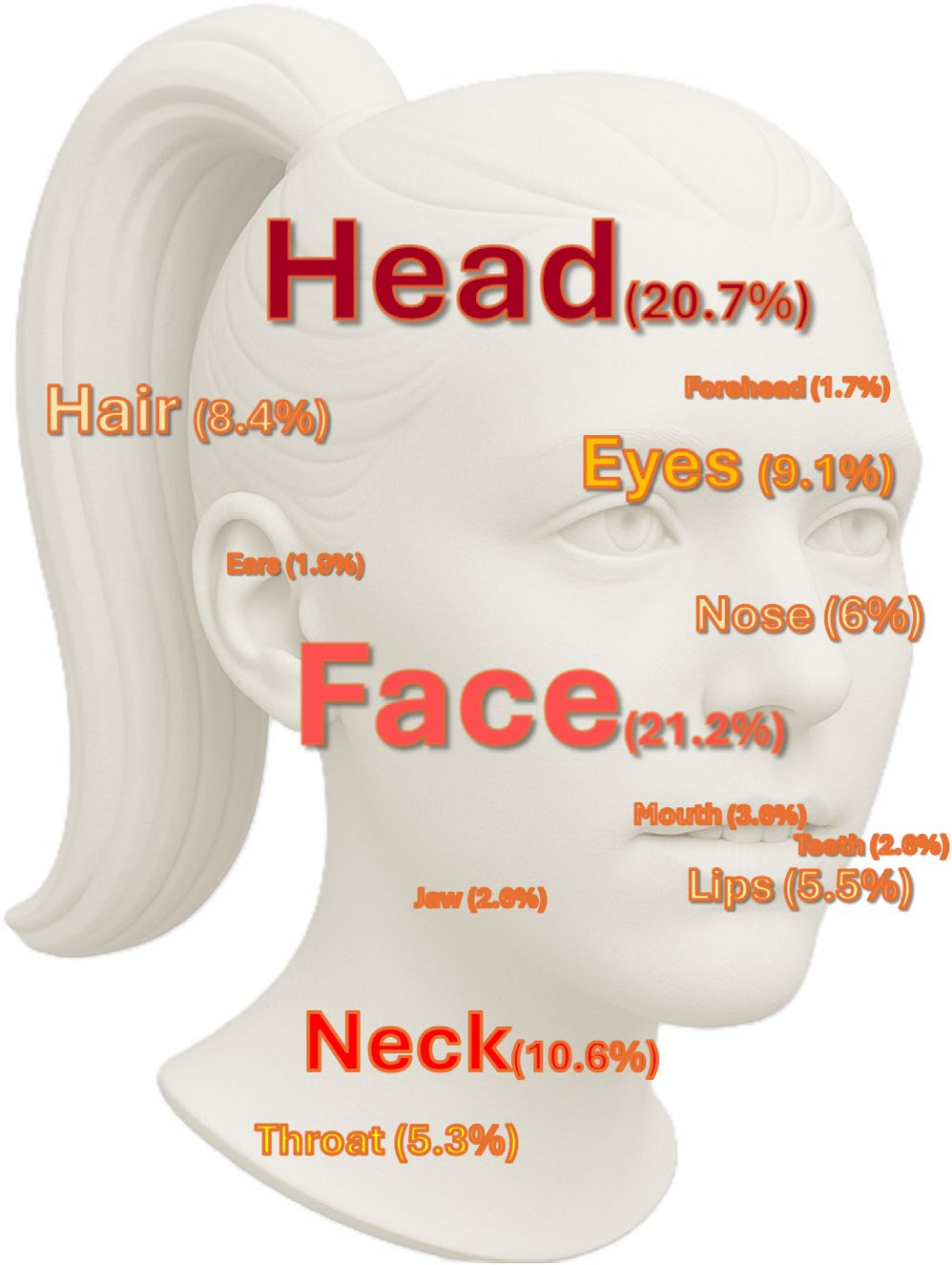
Distribution of injuries by anatomical region (%).

Coded data indicated that the face and head were the most frequently reported sites of injury, followed by the neck, throat, eyes, and hair. Additional affected areas included the nose, lips, mouth, jaw, teeth, ears, forehead, tongue, and temple. A detailed distribution of these injury locations is presented in Figure 1.

In addition to anatomical references, the data included descriptions of physical actions associated with the reported injuries. The most commonly described actions were slapping or smacking, grabbing, and incidents resulting in bruising or broken structures. Other frequent actions included strangulation, pulling or dragging, slamming, and shoving. Less commonly mentioned behaviors included pushing, punching, throwing, and bashing. The least reported were kicking, head‐butting, and hitting with a fist. These actions and their relative frequencies are presented in Table 1. These findings indicate a high prevalence of both blunt‐force trauma and restraint‐type behaviors, with particular concern regarding the frequency of strangulation, given its strong association with serious injury caused by DA.

**TABLE 1:** Frequency of physical actions reported.

Action	Count	%
Slap/smack	44	18
Grab	41	16.8
Bruise/broken	40	16.4
Strangle/choke	36	14.8
Pull/drag	25	10.2
Slam	23	9.4
Shove	14	5.7
Push	7	2.9
Punch	5	2
Throw/threw	4	1.6
Bash	2	0.8
Kick	1	0.4
Head‐butt	1	0.4
Fist	1	0.4

Qualitative analysis of the 153 retained posts revealed four major themes related to DA‐related maxillofacial and dental trauma: (1) Visible injuries, (2) barriers to medical and dental care, (3) psychological and emotional consequences, and (4) healthcare and legal systems. Table 2 provides representative quotes from Reddit users describing lived experiences of domestic abuse in the different themes.

**TABLE 2:** Representative quotes from Reddit users describing lived experiences of domestic abuse.

**1. Visible injuries**
*He's pushed me before (and denies it), but tonight, he actually hit me. He hit me and pushed me so hard that I fell really hard into a night table, and a heavy lamp knocked into my head…*
*My Bf beat me brutally yesterday. I have a knot on my forehead, bruises everywhere, a chipped tooth, and I believe I have a concussion…*
*My ex knocked out some of my front teeth the day I had my baby…*
*When I look at my face, sometimes I see the nose he gave me when he punched me, the crooked smile he gave me when he broke the skin of my cheek, and the scar near the corner of my left eye…*
*…He knocked my teeth right out of my mouth, broke my jaw in two places…*
*…He held my neck by choking me, and shoving his fingers in my mouth… mouth, swollen, black and blue… tongue is swollen with lacerations… I cannot stick my tongue out. I cannot swallow…*
*…His primary form of abuse was punching, full force… I've had dozens of concussions, black eyes, cracked ribs, broken knuckles… He broke the shower tiles with my skull*.
*“He hit me so hard my front tooth chipped. I couldn't go to work like that, so I told them I fell on the ice.”*
*“I had a black eye and a swollen jaw. I used concealer for a week and wore sunglasses indoors.”*
**2. Barriers to medical and dental care**
*I went to the dentist and have not been there in over 10 years… she said to me that my X‐rays reveal everything… she continued to talk to me about resources… I'm praying she hasn't reported it with address. I'm so scared*.
*I haven't been to the dentist in years. He controls all the money and says I don't need it unless I lose a tooth*.
*I wanted to go to the ER when he broke my nose, but I knew they'd ask questions I wasn't ready to answer*.
**3. Psychological and emotional consequences**
*…Today he strangled me. I am still in shock. I couldn't breathe. I was banging on the dresser to get him to let go of me…*
*…I am feeling so hopeless and alone right now. My bf choked me last night unprovoked… I figured I'd call the police which I've never done…*
*My ex knocked out some of my front teeth… I forgot until I was making small talk and laughed and saw my own teeth in a mirror at the store and it just crushed me…*
*…There is a better life but it takes planning and preparing every single detail… I hope you all get out of the situation it's so hopeless to be there*.
*I don't even recognize myself in the mirror anymore. My mouth is swollen, my face is bruised, and I feel disgusting*.
**4. Healthcare and legal systems**
*My girlfriend got arrested last night after I called the cops on her for pulling me out of the car by my hair and then kicking me in the head…*
*…It got so bad to the point where I figured I'd call the police, which I've never done…*
*The dentist asked what happened to my tooth. I said I bit into something hard. He nodded and moved on*.
*The nurse looked at my face and said, ‘Are you safe at home?’ I said yes. She didn't press. I wish she had*.

### 3.1 Theme One: Visible Injuries

Survivors frequently described visible injuries to the head, face, and mouth as a result of physical abuse, using Reddit to disclose, process, and seek validation for these experiences. Posts detailed acts such as being hit, pushed, or thrown against objects, leading to concussions, chipped and knocked‐out teeth, facial bruising, jaw fractures, and swollen or lacerated tongues. The face and mouth emerged as central sites of trauma, often symbolizing the silencing and visibility of abuse. Some victims described the social implications of these injuries, such as missing work or hiding bruises with makeup and sunglasses.

### 3.2 Theme Two: Barriers to Medical and Dental Care

These quotes center on the barriers to accessing medical and dental care caused by a mix of fear, financial control, and safety concerns. Individuals may avoid seeking care because of the fear of being judged or reported, especially if their circumstances might expose them to unwanted scrutiny, such as legal or social consequences. Financial control by a partner or lack of autonomy over money can prevent individuals from accessing necessary care, whereas concerns about personal safety, especially in abusive situations, can deter someone from seeking emergency medical attention, as they fear being questioned or exposed.

### 3.3 Theme Three: Psychological and Emotional Consequences

This theme reveals the psychological impact of abuse on survivors, particularly how it affects their emotional well‐being and sense of self. Survivors often experience feelings of fear, hopelessness, self‐alienation, and trauma, which persist long after physical injuries have healed. Physical violence, especially strangulation and facial injuries, leaves lasting marks not only on the body but also on the survivor's mental health and self‐perception. Triggers, like seeing their own reflection, can bring back intense emotional responses, reigniting past trauma. Survivors also commonly report isolation, powerlessness, and emotional numbness.

### 3.4 Theme Four: Healthcare and Legal Systems

The victims often shared their struggles with the healthcare and legal systems, revealing how difficult it is to find support or understanding when seeking help. Many described encounters with medical professionals or law enforcement where their injuries or abuse were overlooked or not properly addressed. Some survivors expressed frustration at missed opportunities for intervention, such as when doctors or nurses failed to ask the right questions or follow up on signs of abuse.

## 4 Discussion

The present study used web scraping as a methodological tool to access narratives of DA shared by victims and survivors in online spaces. Reddit, a widely used platform for community dialogue, offered an opportunity to explore unfiltered expressions of trauma, particularly those involving orofacial injuries. Traditional data collection methods, such as interviews in clinical settings or analysis of patient records, present significant limitations for DA research, especially when focusing on injuries to the head, neck, and oral regions [4, 24]. Victims often do not disclose abuse during medical or dental appointments because of fear, shame, or a lack of trust in professionals [24, 25, 26]. Ethical concerns about revictimization, as well as the difficulty of reaching survivors during or shortly after abusive incidents, further complicate in‐person data collection. Web scraping might be helpful in overcoming some of these barriers by enabling the collection of firsthand anonymous accounts shared in the public domain and preserving the authenticity of victims/survivors' voices. Reddit's structure supports thematic and anonymous discussions, making it particularly suitable for sensitive topics like interpersonal violence.

The innovation of this study focuses on digital narratives describing oral and maxillofacial injuries and related health‐seeking behaviors among domestic abuse victims/survivors. Although prior studies have examined DA in clinical settings [4, 9], few have employed online and public methods to analyze narratives specific to injuries affecting the head, neck, and oral regions. These injuries are clinically significant as they might serve as red flags for abuse; yet, they are often hidden by the victims or overlooked by healthcare providers [5, 8]. Moreover, the current analysis captures a broader spectrum of victims/survivors' experiences by including diverse gender identities and relational contexts. The integration of web scraping with qualitative content analysis represents a novel contribution to both public health and social research, allowing for the real‐time examination of stigma, access to care, and systemic responses.

The quantitative findings presented in Table 1 and Figure 1 underscore both the severity and anatomical specificity of trauma experienced by DA survivors, particularly in regions such as the face, head, and oral cavity. The prominence of facial, oral, and neck injuries, along with the frequent descriptions of physical actions such as slapping, grabbing, and strangulation, highlights the need for dental professionals to be better equipped to recognize potential signs of abuse. Although prior research has documented the prevalence of orofacial injuries in clinical DA cases, this study adds new insight into how victims/survivors describe such experiences in digital spaces, often revealing both physical harm and barriers to seeking care.

In this study, the thematic analysis revealed four dominant patterns across the Reddit posts. First, survivors described *visible injuries*, such as bruises, broken teeth, and concussions, often located in the face and head. These injuries were not only painful but also sources of shame and fear, prompting efforts to conceal them through makeup, clothing, or deception. Many survivors expressed hesitation in seeking care, fearing that disclosure might provoke retaliation or a further loss of autonomy. These reports support existing findings on the psychological impact of abuse and the strategies victims adopt to protect themselves, both physically and emotionally [11, 27].

Second, there were significant reports on *barriers to accessing both medical and dental car*e. Survivors reported that their abusers often exerted financial or logistical control, preventing them from scheduling appointments or affording treatment. Even when care was accessed, fear of mandatory reporting or judgmental responses from professionals discouraged full disclosure. For instance, some survivors recounted visiting dentists who noticed their injuries but either failed to inquire further or did so in a superficial or dismissive way. These findings align with previous studies evaluating dentists' knowledge about DA, which argue that dental professionals need better training to identify and respond to signs of DA [7, 8].

Third, the *psychological and emotional consequences* of DA emerged as a central theme in survivors' narratives, revealing how trauma extends far beyond physical injuries. Many individuals described feelings of shock, fear, hopelessness, and profound isolation, often accompanied by emotional numbness and a fragmented sense of self. Visible injuries, particularly to the face and mouth, intensified these effects by disrupting self‐image and serving as constant reminders of the abuse. For some, looking in the mirror became a source of re‐traumatization, reinforcing internal suffering through external evidence of violence. The visibility of oral and maxillofacial injuries, which are difficult to hide, contributed to heightened shame and social withdrawal, further isolating survivors from support systems. Additionally, the chronic stress of navigating unsafe environments left many emotionally exhausted, with several expressing a desire to escape but acknowledging the immense emotional and logistical challenges involved in leaving. These narratives underscore the deep psychological toll that abuse inflicts, illustrating how trauma is not only physically embodied but also relived daily through fear, altered identity, and persistent emotional distress [11, 28, 29].

Finally, interactions with the *healthcare and legal systems* were described as inconsistent. Although a few healthcare workers offered support or resources, others overlooked clear signs of violence or failed to follow up after initial questioning. Similarly, some survivors viewed police intervention as a last resort, citing past experiences of being ignored or retraumatized. These findings suggest an urgent need for trauma‐informed practices and standardized training across the healthcare and law enforcement sectors. Effective interventions begin with active listening and nonjudgmental inquiry [15], two elements that were frequently absent in the narratives analyzed in this study.

It is noteworthy that Reddit users come from a wide range of countries and backgrounds, and the legal, social, cultural, and economic contexts can vary significantly across regions and countries. Some countries might have strong legal protections and support systems in place for victims of domestic violence, whereas others might lack both legal safeguards and cultural support. Similarly, economic barriers such as access to healthcare can differ widely; in some countries, medical services related to abuse may be fully covered by insurance or government programs.

The results of this web scraping highlight a clear and repeated pattern of violence involving areas that are often within the clinical scope of dental professionals. The concentration of injuries to the face, mouth, and neck, coupled with the high frequency of violent actions targeting these regions, suggests that dental professionals are uniquely positioned to identify signs of DA early.

This opportunity is frequently missed because of a lack of training, discomfort with initiating conversations about abuse, or unawareness of referral pathways. The knowledge generated from this study underscores the importance of the systematic integration of DA screening and trauma‐informed care into dental education, clinical protocols, and continuing professional development. Recognizing patterns of injury and their broader psychosocial context empowers oral health professionals to contribute meaningfully to multidisciplinary efforts aimed at identifying, supporting, and protecting DA victims/survivors. This study also demonstrates the value of ethical digital research methods, such as web scraping, as a complementary approach to traditional data collection, especially when working with this sensitive population.

## Author Contributions

All authors made substantial contributions to the manuscript. This includes conceptualization, methodology, validation, investigation, resources, writing the original draft, reviewing, and editing. All authors have read and approved the final version of the manuscript.

## Ethics Statement

The study was exempt from review and approval by the Research Ethics Board (REB) at the University of Saskatchewan (E‐Bio‐060).

## Conflicts of Interest

The authors declare no conflicts of interest.

## Data Availability

The data that support the findings of this study are available from the corresponding author upon reasonable request.
